# Feasibility, tolerability and efficacy of the ketogenic diet in children with drug‐resistant epilepsy in Vietnam

**DOI:** 10.1002/epi4.12825

**Published:** 2023-10-03

**Authors:** Thuy Minh Thu Nguyen, Pierre Jallon, Christian Korff, Hieu Nguyen, Sylvie Nguyen The Tich

**Affiliations:** ^1^ Department of Pediatric University of Medicine and Pharmacy at Ho Chi Minh City Ho Chi Minh City Vietnam; ^2^ Deparment of Neurology Children's Hospital 2 Ho Chi Minh City Vietnam; ^3^ Department of Neurology University of Medicine and Pharmacy at Ho Chi Minh City Ho Chi Minh City Vietnam; ^4^ Pediatric Neurology Unit Geneva University Hospitals Geneva Switzerland; ^5^ Faculté de Médecine Henri‐Warembourg Université du Droit et la Santé de Lille 2 Lille Centre Hospitalier Régional Universitaire de Lille Lille France

**Keywords:** children, feasibility, ketogenic diet, tolerability, Vietnam

## Abstract

**Objectives:**

According to the WHO, more than 50 million people have epilepsy. Among them, nearly 80% of patients with epilepsy live in developing countries and 75% of them do not have access to treatment. The ketogenic diet (KD) has been shown as an effective alternative for patients with drug‐resistant epilepsy. Although it has been studied in Asia, no such studies have been conducted in Vietnam. The purpose of this study was to verify the feasibility and tolerability of KD in children with refractory epilepsies in Vietnam.

**Methods:**

Children with drug‐resistant epilepsy followed at Children's Hospital, Vietnam treated by KD were included in a prospective study from June 2019 to October 2021. Side‐effects, retention rate, number, and duration of seizures were recorded after 1, 3, 6, 9 and 12 months of KD. Patients were considered as respondents when a 50% seizure frequency was reached. Tolerance and acceptability of the KD were closely monitored.

**Results:**

Forty‐six children were included but KD was contraindicated for one patient. Due to the COVID pandemic, we had to rely on internet exchanges to stay in touch with families. Meals had to be adapted to Vietnamese culinary habits. The retention rate decreased from 82.2% at 1 month to 40% at 12 months of follow‐up. The incidence of side effects was 44.4% and occurred mainly during the first month. Fifteen patients out of 45 were considered as responders after 12 months.

**Significance:**

Our study was the first attempt to introduce KD in Vietnam. It demonstrated that this diet was feasible and well tolerated. The KD diet resulted in significant improvement for 30% of our patients with drug‐resistant epilepsy. This percentage is lower than in some studies but warrants the use of KD as a valuable alternative in a country where many patients lack access to recent treatments.

**Key points**

- Ketogenic diet was largely considered as feasible in a group of children with pharmacoresistant epilepsies in Vietnam.
- After 1 year, the ketogenic diet appeared to be well tolerated and led to significant improvement for a third of the patients. However, side effects were reported by less than half of the patients.
- Ketogenic diet represents a valuable alternative for patients with drug‐resistant epilepsy in Vietnam.

## 1 INTRODUCTION

According to the World Health Organization, more than 50 million people worldwide have epilepsy. Among them, nearly 80% of patients with epilepsy live in low‐ and middle‐income countries and 75% of them do not have access to treatment.^1^ In this part of the world, the unavailability and high prices of anti‐seizure medications along with limited access to treatment options, such as epilepsy surgery and vagus nerve stimulation, lead to a significant treatment gap within the population.

In Vietnam, one study in a rural district described an incidence of epilepsy around 40 per 100 000 with an 84.7% treatment gap.^2^ The reasons for stopping treatment were the cost of treatment (5.3% of patients), locally unavailable drug (10.5% of patients) and the feeling that treatment was too expensive for a low seizure frequency. The incidence of epilepsy and treatment gap were highest in children (67 per 100 000 and 93%, respectively).^2^ In Vietnam, phenobarbital and phenytoin are delivered free of charge; carbamazepin, valproate, diazepam, levetiracetam and topiramate are also accessible.^3^ However, for certain patients who live in rural areas, the distance between their home and healthcare facilities also contributes to the treatment gap.

Moreover, some of these treatments are not effective or contraindicated in some childhood epileptic syndromes.^4^, ^5^ Finally, other recommended medications like stiripentol and clobazam in children with Dravet syndrome are not available in Vietnam, except for wealthy families who can buy them from the Internet.

For the numerous patients with severe drug‐resistant seizures, the ketogenic diet (KD) could be a valuable alternative. KD has been shown to be as effective and used in some Asian countries such as India, Korea, China and Thailand.^6^, ^7^, ^8^, ^9^ In Vietnam, implementing a high fat‐low carbohydrate diet could be challenging considering that the ordinary regimen is based on rice, a high carbohydrate aliment.

The objective of our study was to evaluate if KD was feasible, safe and effective for children with refractory epilepsy in Vietnam.

## 2 MATERIALS AND METHODS

We conducted a prospective case study at Children's Hospital 2 at Ho Chi Minh City (HCMC) from September 2019 to October 2021. The study was approved by the medical ethics committee of Children's Hospital 2 and registered in Clinical Trial number NCT05697887. Inclusion criteria were as follows: age from 1 to 10 years old at the time of enrolment, drug‐resistant epilepsy according to the 2010 ILAE criteria, and an indication for a ketogenic diet trial according to the 2018 ILAE recommendations.^10^, ^11^ Exclusion criteria were clinical or laboratory features suggesting an underlying disease contra‐indication the use of a KD.

Informed consent was obtained from the family after detailed explanation.

Ketogenic diet was introduced under close medical supervision, either as inpatients or during frequent hospital visits for families living nearby without a fasting period.

An introduction to KD was proposed via two videos made by our team. One explained what the ketogenic diet is, the other one described specific ketogenic recipes. To make cooking easier, we proposed simple recipes including red pumpkin, rice paper, and cooking oil which parents can make in a few minutes (Table 1). We also offered dishes that can be stored for a long time such as spring rolls to help mothers reduce the lack of cooking time. We gave families different recipes with different KD ratios and explained that they could make some changes depending on seizure frequency and the child's appetite. As a result, children did have a KD ratio that may vary from day to day. A modified Atkins diet was proposed as an alternative option when necessary. Vitamins were added to the diet as recommended.^12^

**TABLE 1:** Examples of ketogenic diet menus.

Recipe	Ingredients	Instructions	Calories and L/G ratio
Pumpkin soup	30 g pumkin 20 g porridge 20 g pork 7.5 g medium triglyceride power 8 g cooking oil (ratio 1:1), or 20 g cooking oil (ratio 2:1), or 35 g cooking oil (ratio 3:1) or 50 g cooking (ratio 4:1)	Peel, wash and finely chop the pumpkin. Next, steam the pumpkin and cook it with porridge Wash and mince the meat When the porridge and pumpkin are boiling, add the meat and cook for another 5 min Put the meat porridge and pumpkin mixture down. When the mixture cools, add in the mixture of medium chain triglycerides and cooking oil. Stir well to combine the cooking oil and medium chain triglycerides into the mixture Enjoy	175 kcal, ratio 1:1 283 kcal, ratio 2:1 418 kcal, ratio 3:1 553 kcal, ratio 4:1
Traditional Vietnam sping roll	4 rice papers 30 g chicken 30 g boiled pork fat, diced, air‐dried 20 g onion 1 egg yolk 25 g mayonnaise (ratio 3:1) or 50 g mayonnaise (ratio 4:1) Lettuce, herbs (optional)	To make the filling, mix minced meat, onion, lard, egg yolk Put the filling in the pie shell, roll it up, and seal the edge with egg white Turn on low heat and fry the spring rolls until golden brown Serve spring rolls with salad with herbs of your choice, dipping mayonnaise	600 kcal, ratio 3:1 780 kcal, ratio 4:1

The patients had to keep two diaries, one for seizure data and the other for food in which parents wrote down intakes on a daily basis. For illiterate parents, we kept in contact by phone call, asking them about food and seizures. Parents were invited to follow our Facebook page where they could discuss with other families.

Follow‐up visits were planned at 1, 3, 6, 9 and 12 months after the KD started. These visits were done in person or through video calls and social media messages when the patients could not attend in person due to social distancing related to the SARS‐CoV2 pandemics.

At each follow‐up visit, the nutrition team checked the food diary in order to estimate the real KD ratio and fat, carbs and protein intakes. We evaluated if the diet was feasible for the families and the occurrence of side effects. Tolerability was defined as the absence of any side effect as per the parent's report. The number and duration of seizures, as well as the occurrence of status epilepticus, were noted. Status epilepticus was defined as seizures objectively lasting more than 5 minutes and requiring hospitalization.^13^

We assessed the effectiveness of the ketogenic diet based on three criteria: seizure frequency per month, mean duration of seizures, and the absence of status epilepticus. The ketogenic diet was considered as effective if a 50% reduction in the frequency of attacks after the first month was observed.^11^

Qualitative variables are reported as percentages, while quantitative variables were measured as mean (or median) and standard deviation (or interquartile range).

## 3 RESULTS

Forty‐six patients were included. Among them, 1 child had hyperlipidemia at initial workup that contraindicated the start of the KD. The remaining 45 children started on KD. Population characteristics are exposed in Table 2. All children had more than 2 medications and more than 2 seizures types.

**TABLE 2:** Baseline characteristics of the patients at the time of ketogenic diet initiation.

Baseline characteristics	Patients at the time of KD initiation
Age at epilepsy onset in months (mean, median, range)	10.6; 4; 3–11
Age at KD initiation in months (mean, median, range)	41.3; 37; 24–51.5
Number of ASMs failed before the initiation of KD (mean)	4.4
Male/female (n)	22/23
Average seizure duration in minutes (mean, median, range)	Mean 7.6, median 3 (1–6.5)

Thirty‐six children lived in South Vietnam and nine in other regions. One‐third (15/45) of children lived in families with an income of less than $600 a year per person. Four came from illiterate families.

The median age at epilepsy onset was 4  months.^3^, ^4^, ^5^, ^6^, ^7^, ^8^, ^9^, ^10^, ^11^ Patients received four different types of medication on average before being put on the ketogenic diet. The median time from the child's first seizure to KD start was 27 [15–36] months.

Nine patients (eight patients with Dravet syndrome with SCN1A mutations and one patient with Rasmussen encephalitis) were included because of convulsive status epilepticus needing hospitalization. One patient with Landau Kleffner syndrome and GRIN2A mutation was included after being resistant to corticoids, clobazam, and valproic acid. Clobazam was bought from abroad by the family. Fifteen (33%) had drug‐resistant spasms (including 13 patients with West syndrome, and two patients with Ohtahara syndrome). Twenty patients presented focal seizures as their main symptoms, including 16 with known etiology and four with progressive epileptic encephalopathy of unknown etiology. In this group, one patient had a SMC1A mutation that had been previously described.^14^ She had her first seizure at 19 months of age. At the time of admission to the diet, her seizures were intractable to multiple anti‐seizure medications. The diet change resulted in a 6‐month seizure‐free period.

KD was started as in‐patients for 38 patients. The mean hospital stay was 11.1 days (±9.9 days). The remaining seven patients, who started the diet as outpatients, lived near the hospital and had hospital visits planned every 2–3 days with phone contact possible between visits.

The retention rates at 1, 3, 6, 9, and 12 months were 82.2% (37/45); 75.6% (34/45); 55.6% (25/45); 48.9% (22/45); 40% (18/45), respectively. The main reasons for stopping the KD in the first 3 months were parental disbelief in effectiveness (n = 6), inconvenience (mostly due to time for cooking), or side effects (n = 7). In this group, we noted that the compliance group (n = 37) had a longer hospital stay (initiated in hospital, n = 34; mean 10.1 days) than the early drop‐out group (n = 8, initiated in hospital, n = 4; mean 4.7 days), but the difference was not statistically significant (*P* = 0.058).

Between the third and the sixth month, the main reasons were child refusal to eat (n = 7) and no improvement of epilepsy (n = 5).

Side effects occurred mostly in the first 6 months and were mild but led to five dropouts. The main side effects were gastrointestinal (vomiting 21%, abdominal pain 8%, and weight loss 34%) and were observed during the first month of KD. Ten mothers had to quit their jobs to cook for their children. For children who stayed on KD after the 6th month, KD was well tolerated with weight loss as the only side effect for 8% of patients. Only two patients who were still on KD after 12 months lost weight but their initial BMIs were above 2 (2.8 and 5.6). This weight loss was not considered as a side effect. One patient had hyperlipidemia and one had hypoalbuminemia under the 4:1 KD diet. These patients switched to the modified Atkins diet, and hyperlipidemia as well as hypoalbuminemia were improved 1 month later.

One girl with Dravet syndrome who was on the diet for 9 months died suddenly at 43 months. Her mother put her to bed after a normal day and found her dead the next morning. Before KD, she had monthly status epilepticus episodes (lasting 20 minutes to 2 hours) that needed hospitalization. With the KD, she had more frequent but shorter seizures without needing to be hospitalized. This girl was receiving four anti‐seizure medications (valproic acid, topiramate, levetiracetam, and diazepam) in addition to the diet. She had no severe side effects except for light vomiting in the first month. No autopsy was performed.

**FIGURE 1:**
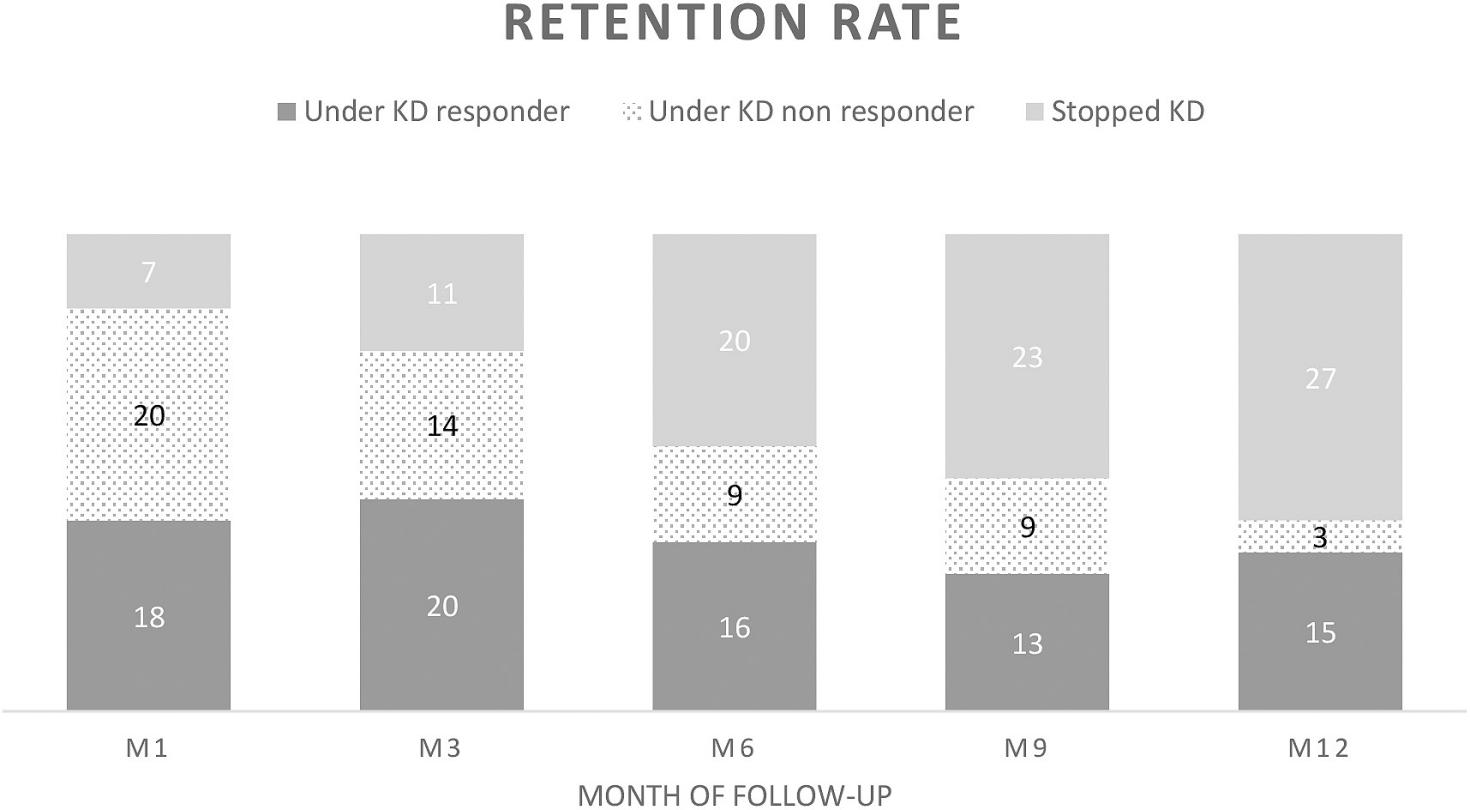
Retention rate by month of follow‐up.

After one‐year follow‐up, 15 patients were considered as responders and five children were seizure‐free, including two patients with Dravet syndrome, one patient with unknown etiology West syndrome and two in the focal seizure group (tuberous sclerosis complex and cortical malformation; Figure 1). The effects of KD on seizure frequency and seizure duration are shown in Table 3.

**TABLE 3:** Evolution of seizure number and seizure duration by month of follow‐up.

Patients under KD	M0 (n = 45)	M1 (n = 38)	M3 (n = 34)	M6 (n = 25)	M9 (n = 22)	T12 (n = 18)
Median seizure number per month [25–75 IQR]	60 [5.5–205]	37.5 [4.5–120]	4 [0–92.5]	10 [0.6–70]	6.5 [0.2–90]	2 [0–16.5]
Median seizure duration in minute [25–75 IQR]	3 [1–6.5]	1.25 [0.2–4]	0.5 [0–4]	1 [0.2–4]	0.4 [0.1–3.25]	0.2 [0.1–1.2]

Abbreviation: KD, ketogenic diet.

## 4 DISCUSSION

In our study on 45 children with drug‐resistant epilepsy, KD was feasible and well tolerated. One third of our patients responded to the diet with five children out of 45 being seizure free.

KD was achievable low‐income families (income less than 600 USD a year per person) by using low‐priced, locally available fat‐rich products, such as coconut oil or avocado. However, to vary the menus and encourage the child to eat, some families had to buy meat that was too expensive and resulted in the KD being abandoned. Despite simplified recipes, 10 mothers had to quit their jobs to allow time for meals preparation in accordance with KD. Ketogenic products and recipes are outside the norm of regularly available food in Vietnam. For illiterate patients, cooking videos made available on social media were helpful.

Keeping in touch with parents has been demonstrated to be crucial to a successful diet.^15^ Currently, many Vietnamese people use smartphones and social media, so we contacted them through the Internet. When patients had any specific questions about the ketogenic diet, they could easily stay in touch with our team via a Facebook group. Parents could also suggest new recipes. We verified the ratio before publication. We currently have more than 50 recipes.

Almost half of the patients had side effects (44.4%), all of which were mild. The most common ones were gastrointestinal symptoms (weight loss, vomiting, abdominal pain) and were most frequent during the first month. The incidence of adverse events in our study was similar to that reported by Ruiz Herrero (42%) and Geres (54%).^16^, ^17^ However, we did not report adverse effects, such as hypoglycemia or severe dehydration, leading to acute renal failure. We provided close in‐hospital monitoring during the first day for most of our patients. Therefore, they were treated immediately from the first symptom (vomiting, abdominal pain…). One patient had hyperlipidemia and one patient had hypoalbuminemia while they applied the traditional 4:1 diet. These patients were successfully switched to the modified Atkins diet.

Many families were unable to return to regular check‐ups due to distance and to Covid travel restriction. Consequently, no follow up tests were possible during the Covid pandemic.

The retention rate was 40% after 12 months. This rate is similar to the one reported in the study by Gerges et al^17^ The high expectation of some parents regarding the efficiency of the KD led them to abandon out of disappointment. They expected more than a 50% seizure reduction to consider it as effective. In the Vietnamese prevalence study, one of the main reasons for patients to drop medication was a low expected benefit with respect to seizure burden.^3^

The proportion of responders fluctuated over time. The response rates tended to decrease slightly after the 3rd month. Some patients cannot fully comply with treatment for a long time (loss of appetite, distasteful food). It is possible that some patients may have taken medicines containing sugar. Even though this has not been proven, it may have reduced the effectiveness of the diet.

Half of our eight patients with Dravet syndrome saw a significant improvement in seizure duration with a decreased rate of hospitalization. This is of particular interest in our country as patients have no or little access to usually recommended drugs (stiripentol, clobazam, buccal midazolam) in this specific syndrome. This result is in accordance with recent studies and recommendations and led us to adapt our practice for these patients.^18^

## 5 CONCLUSION

Through this study, we demonstrated that KD is feasible and safe in Vietnam, a low‐income country with limited medical resources and whose traditional food is rice. To implement this diet, locally available food sources can be used at low and acceptable costs. To be successful with the diet, health workers (child neurologists and dietitians) need to closely follow up patients. Modern techniques, such as social networks, websites and e‐health applications, were essential for us to monitor this treatment.

## AUTHOR CONTRIBUTIONS

Thuy Minh Thu Nguyen: Analysis of literature, design of the study, data analysis, and writing and approval of the manuscript. Pierre Jallon: writing and approval of the manuscript. Christian Korff: writing and approval of the manuscript. Hieu Nguyen: Analysis of literature, design of the study. Sylvie Nguyen The Tich: Analysis of literature, design of the study, writing and approval of the manuscript. Maï‐Ly Dubreuil English editing.

## CONFLICT OF INTEREST STATEMENT

Thuy Minh Thu Nguyen has received support from the FFRE (Fondation Française pour la Recherche sur l'Epilepsie). Sylvie Nguyen The Tich is a member of the FFRE scientific committee. Neither of the other authors has any conflict of interest to disclose. We confirm that we have read the Journal's position on issues involved in ethical publication and affirm that this report is consistent with those guidelines.

## ETHICS STATEMENT

The study was approved by the Ethics Council in a biomedical study at Children's Hospital 2.

## PATIENT CONSENT STATEMENT

I understand that my participation is voluntary and that I am free to withdraw at any time, without giving a reason and without cost. I understand that I will be given a copy of this consent form. I voluntarily agree to take part in this study.

## CLINICAL TRIAL REGISTRATION

ClinicalTrials.gov Identifier: NCT05697887.

## Data Availability

The data that support the findings of this study are available from the corresponding author upon reasonable request.

## References

[1] World Health Organisation . https://www.who.int/health‐topics/epilepsy#tab=tab_1. Consulted December 20, 2023.

[2] Tuan NA , Tomson T , Allebeck P , Chuc NTK , Cuong LQ . The treatment gap of epilepsy in a rural district of Vietnam: a study from the EPIBAVI project. Epilepsia. 2009;50(10):2320–2323. 19744115 10.1111/j.1528-1167.2009.02298.x

[3] Mac TL , Le VT , Vu AN , Preux PM , Ratsimbazafy V . AEDs availability and professional practices in delivery outlets in a city center in southern Vietnam. Epilepsia. 2006;47(2):330–334. 16499757 10.1111/j.1528-1167.2006.00425.x

[4] Cardenal‐Muñoz E , Auvin S , Villanueva V , Cross JH , Zuberi SM , Lagae L , et al. Guidance on Dravet syndrome from infant to adult care: road map for treatment planning in Europe. Epilepsia Open. 2022;7(1):11–26. 34882995 10.1002/epi4.12569 PMC8886070

[5] Wilmshurst JM , Gaillard WD , Vinayan KP , Tsuchida TN , Plouin P , van Bogaert P , et al. Summary of recommendations for the management of infantile seizures: task force report for the ILAE Commission of Pediatrics. Epilepsia. 2015;56(8):1185–1197. 26122601 10.1111/epi.13057

[6] Li H , Ouyang M , Zhang P , Fei L , Hu X . The efficacy and safety of a ketogenic diet for children with refractory epilepsy in China: a retrospective single‐center cohort study. Transl Pediatr. 2020;9(4):561–566. 32953554 10.21037/tp-20-219 PMC7475313

[7] Chomtho K , Suteerojntrakool O , Chomtho S . Effectiveness of medium‐chain triglyceride ketogenic diet in Thai children with intractable epilepsy. J Med Assoc Thai. 2016;99(2):159–165. 27249895

[8] Kang HC , Kim YJ , Kim DW , Kim HD . Efficacy and safety of the ketogenic diet for intractable childhood epilepsy: Korean multicentric experience. Epilepsia. 2005;46(2):272–279. 15679508 10.1111/j.0013-9580.2005.48504.x

[9] Sharma S , Gulati S , Kalra V , Agarwala A , Kabra M . Seizure control and biochemical profile on the ketogenic diet in young children with refractory epilepsy—Indian experience. Seizure. 2009;18(6):446–449. 19427240 10.1016/j.seizure.2009.04.001

[10] Kossoff EH , Zupec‐Kania BA , Auvin S , Ballaban‐Gil KR , Christina Bergqvist AG , Blackford R , et al. Optimal clinical management of children receiving dietary therapies for epilepsy: updated recommendations of the international ketogenic diet study group. Epilepsia Open. 2018;3(2):175–192. 29881797 10.1002/epi4.12225 PMC5983110

[11] Kwan P , Arzimanoglou A , Berg AT , Brodie MJ , Allen Hauser W , Mathern G , et al. Definition of drug‐resistant epilepsy: consensus proposal by the ad hoc Task Force of the ILAE Commission on Therapeutic Strategies. Epilepsia. 2010;51(6):1069–1077. 19889013 10.1111/j.1528-1167.2009.02397.x

[12] Kossoff EH , al‐Macki N , Cervenka MC , Kim HD , Liao J , Megaw K , et al. What are the minimum requirements for ketogenic diet services in resource‐limited regions? Recommendations from the international league against epilepsy task force for dietary therapy. Epilepsia. 2015;56(9):1337–1342. 26033161 10.1111/epi.13039

[13] Trinka E , Cock H , Hesdorffer D , Rossetti AO , Scheffer IE , Shinnar S , et al. A definition and classification of status epilepticus – report of the ILAE Task Force on Classification of Status Epilepticus. Epilepsia. 2015;56(10):1515–1523. 26336950 10.1111/epi.13121

[14] Hieu NLT , Thu NTM , Ngan LTA , Van LTK , Huy DP , Linh PTT , et al. Genetic analysis using targeted exome sequencing of 53 Vietnamese children with developmental and epileptic encephalopathies. Am J Med Genet A. 2022;188(7):2048–2060. 35365919 10.1002/ajmg.a.62741

[15] Costa AM , Marchiò M , Bruni G , Bernabei SM , Cavalieri S , Bondi M , et al. Evaluation of E‐health applications for paediatric patients with refractory epilepsy and maintained on ketogenic diet. Nutrients. 2021;13(4). 10.3390/nu13041240 PMC8069190 33918854

[16] Ruiz Herrero J , Cañedo Villarroya E , García Peñas JJ , García Alcolea B , Gómez Fernández B , Puerta Macfarland LA , et al. Safety and effectiveness of the prolonged treatment of children with a ketogenic diet. Nutrients. 2020;12(2). 10.3390/nu12020306 PMC7071522 31991539

[17] Gerges M , Selim L , Girgis M , el Ghannam A , Abdelghaffar H , el‐Ayadi A . Implementation of ketogenic diet in children with drug‐resistant epilepsy in a medium resource setting: Egyptian experience. Epilepsy Behav Case Rep. 2019;11:35–38. 30619711 10.1016/j.ebcr.2018.11.001 PMC6312833

[18] Yan N , Xin‐Hua W , Lin‐Mei Z , Yi‐Ming C , Wen‐Hui L , Yuan‐Feng Z , et al. Prospective study of the efficacy of a ketogenic diet in 20 patients with Dravet syndrome. Seizure. 2018;60:144–148. 29990707 10.1016/j.seizure.2018.06.023

